# Laser-activated perfluorocarbon nanodroplets for intracerebral delivery and imaging via blood–brain barrier opening and contrast-enhanced imaging

**DOI:** 10.1186/s12951-024-02601-6

**Published:** 2024-06-21

**Authors:** Kristina A. Hallam, Robert J. Nikolai, Anamik Jhunjhunwala, Stanislav Y. Emelianov

**Affiliations:** 1 The Wallace H. Coulter Department of Biomedical Engineering, Georgia Institute of Technology and Emory University School of Medicine, Atlanta, GA USA; 2 School of Electrical and Computer Engineering, Georgia Institute of Technology, Atlanta, GA USA

**Keywords:** Photoacoustic imaging, Ultrasound imaging, Perfluorocarbon nanodroplets, Drug delivery, Neuroimaging, Contrast-enhanced imaging, Multiplex imaging, Blood‒brain barrier opening

## Abstract

**Background:**

Ultrasound and photoacoustic (US/PA) imaging is a promising tool for in vivo visualization and assessment of drug delivery. However, the acoustic properties of the skull limit the practical application of US/PA imaging in the brain. To address the challenges in targeted drug delivery to the brain and transcranial US/PA imaging, we introduce and evaluate an intracerebral delivery and imaging strategy based on the use of laser-activated perfluorocarbon nanodroplets (PFCnDs).

**Methods:**

Two specialized PFCnDs were developed to facilitate blood‒brain barrier (BBB) opening and contrast-enhanced US/PA imaging. In mice, PFCnDs were delivered to brain tissue via PFCnD-induced BBB opening to the right side of the brain. In vivo, transcranial US/PA imaging was performed to evaluate the utility of PFCnDs for contrast-enhanced imaging through the skull. Ex vivo, volumetric US/PA imaging was used to characterize the spatial distribution of PFCnDs that entered brain tissue. Immunohistochemical analysis was performed to confirm the spatial extent of BBB opening and the accuracy of the imaging results.

**Results:**

In vivo, transcranial US/PA imaging revealed localized photoacoustic (PA) contrast associated with delivered PFCnDs. In addition, contrast-enhanced ultrasound (CEUS) imaging confirmed the presence of nanodroplets within the same area. Ex vivo, volumetric US/PA imaging revealed PA contrast localized to the area of the brain where PFCnD-induced BBB opening had been performed. Immunohistochemical analysis revealed that the spatial distribution of immunoglobulin (IgG) extravasation into the brain closely matched the imaging results.

**Conclusions:**

Using our intracerebral delivery and imaging strategy, PFCnDs were successfully delivered to a targeted area of the brain, and they enabled contrast-enhanced US/PA imaging through the skull. Ex vivo imaging, and immunohistochemistry confirmed the accuracy and precision of the approach.

**Graphical Abstract:**

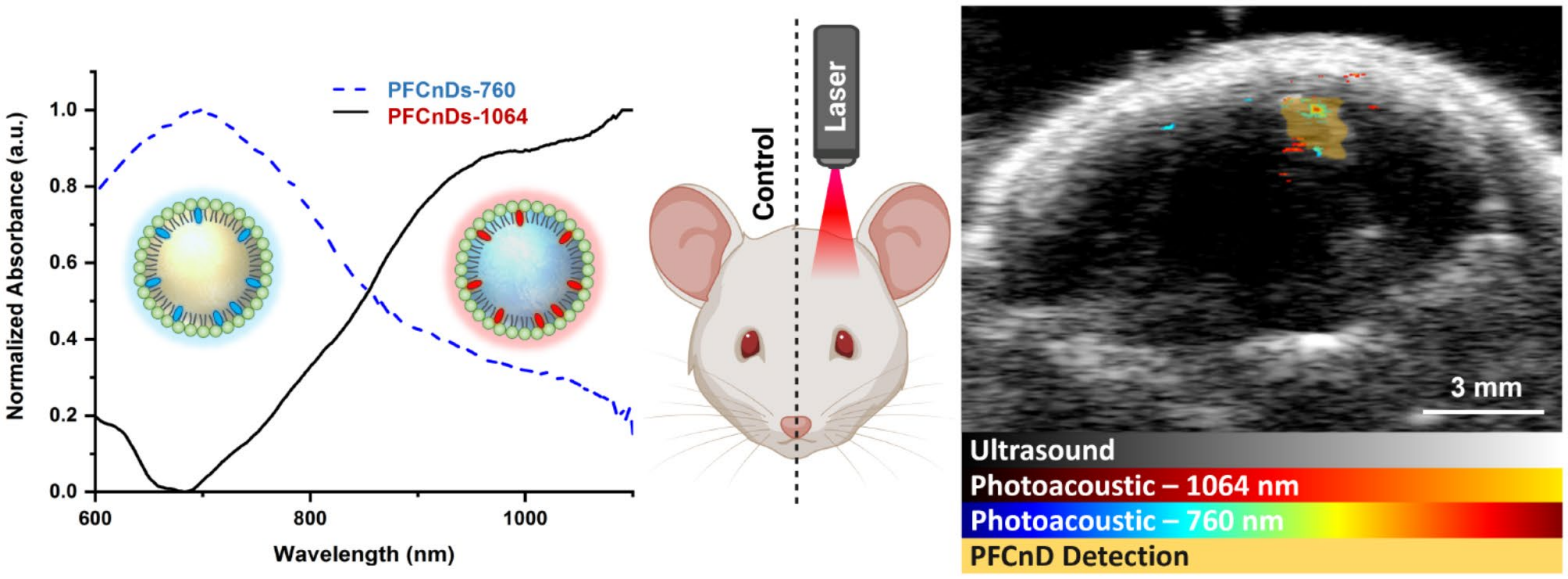

**Supplementary Information:**

The online version contains supplementary material available at 10.1186/s12951-024-02601-6.

## Background

Drug delivery to the brain for the treatment of neurological diseases and disorders still faces significant challenges in overcoming the blood‒brain barrier (BBB) and providing precise targeting. Precision in intracerebral drug delivery is critical because many brain diseases, including Alzheimer’s disease and Parkinson’s disease, have pathologies tied to specific brain regions [1, 2], and off-target drug delivery carries the risk of severe side effects within the brain [3–5]. Current techniques like focused ultrasound (FUS) can disrupt the BBB to aid drug penetration, but there are no approaches to verify whether injected drugs enter the intended brain tissue regions. Therefore, a reliable intracerebral drug imaging and tracking system would offer the clinical benefit of drug delivery verification and localization to aid in effective treatment planning for brain disorders. Combined ultrasound and photoacoustic (US/PA) imaging utilizing specialized imaging contrast nanoagents is a promising method for detailed, longitudinal, and in vivo drug monitoring [6–8]. However, its practical use in clinical neuroimaging is limited by the strong signal dampening and acoustic impedance mismatch of the skull.

Localized BBB opening can be achieved using FUS and microbubbles with MRI guidance [9]. Microbubbles can also mediate transcranial US imaging of cerebral vasculature through contrast-enhanced US (CEUS) imaging. However, due to their large size and short retention time, microbubbles cannot penetrate brain tissue even after BBB disruption. As a result, they cannot provide contrast within brain tissue itself to indicate where delivered therapeutic agents have entered. Magnetic resonance imaging (MRI), despite its utility in neuroimaging, is not ideal for frequent, long-term drug tracking due to its high cost and lengthy acquisition times. By leveraging specialized imaging nanoagents, US/PA imaging could reliably verify successful BBB opening and subsequent penetration of therapeutics into targeted brain regions. US/PA imaging is well suited for drug tracking because it is capable of high-contrast, multiplex, and real-time imaging. It is also nonionizing, cost-effective, and portable, making it a practical tool for longitudinal, point-of-care imaging.

The limitations of microbubbles and traditional imaging modalities in verifying intracerebral drug delivery highlight the need for a new contrast agent capable of mediating BBB opening and enabling verification of successful opening. Perfluorocarbon nanodroplets (PFCnDs) represent a promising solution to these challenges. These biocompatible emulsions of perfluorocarbon liquid and a surfactant shell have diameters of 100–1000 nm, enabling them to penetrate the brain parenchyma following BBB disruption, unlike larger microbubbles, which are 1–10 μm. The key distinctive property of PFCnDs is their ability to be remotely vaporized into PFC microbubbles through acoustic or optical energy simulation expanding their size tenfold [10–14]. Moreover, reversible phase changes allow these PFC microbubbles to recondense back into nanodroplets for repeated use. By customizing their constituent materials, PFCnDs can be tailored for various drug delivery and imaging functions. For instance, they can serve as drug vehicles by carrying molecular cargo and be functionalized with targeting ligands. Additionally, when loaded with an optical trigger/photoabsorber, they can be remotely laser-activated using wavelength-tuned pulses [15]. Finally, through repeated laser-activation, PFCnDs can undergo photoacoustic cavitation which can induce mechanical disruption of nearby tissue [16]. This effect can be used to locally and reversibly open the BBB [17, 18].

PFCnDs offer a unique mode of CEUS imaging with their ‘Boolean’ contrast capability. In their vaporized microbubble state, they provide US contrast, while in their original nanodroplet form, they do not [19]. The state-switching ability of PFCnDs enables precise nanodroplet detection and localization via evaluation of changes in the CEUS signal after vaporization is induced [19–23]. Moreover, laser-activated PFCnDs can generate photoacoustic contrast for multiplex imaging capabilities [24]. We hypothesized that leveraging the intrinsic contrast enhancement of PFCnDs could facilitate transcranial US/PA imaging through the skull for sensitive detection and monitoring of their penetration into brain tissue after BBB opening.

**Scheme 1:**
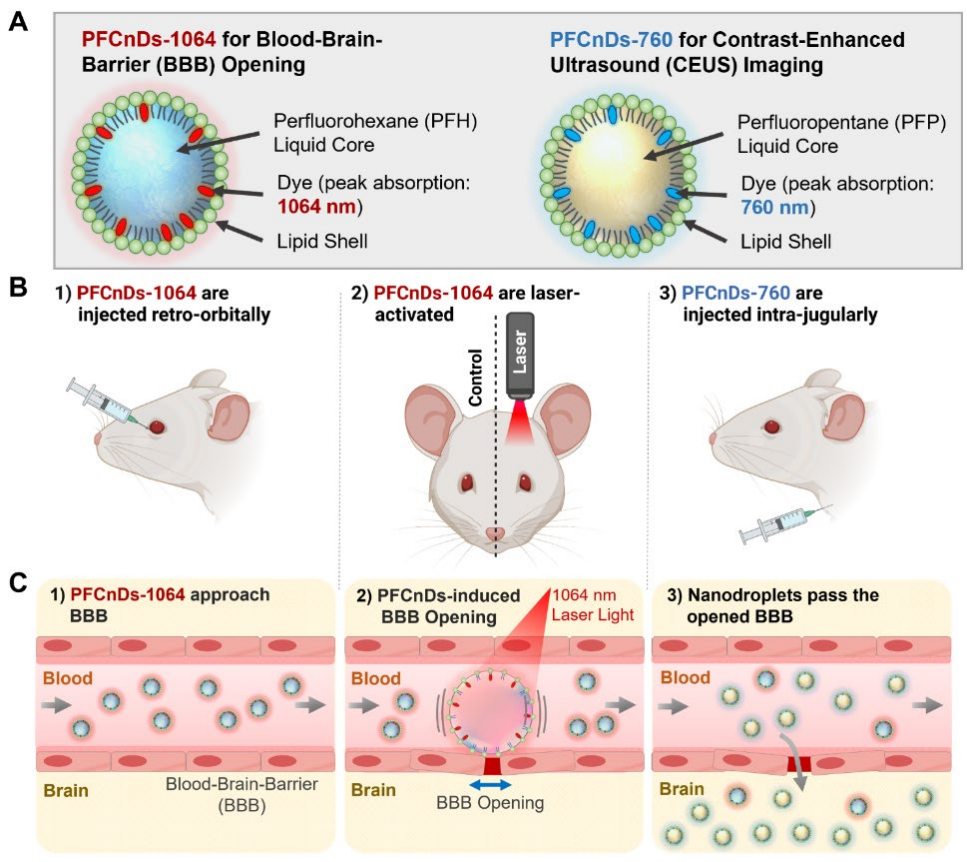
Schematic of the PFCnD-based intracerebral delivery strategy. (**A**) Schematic illustration of the two PFCnDs species for BBB opening and contrast-enhanced US/PA imaging. The core materials were optimized for specific functions with PFH used for BBB opening and PFP used for CEUS imaging. The two nanodroplets species were loaded with optical dyes having peak optical absorbances of 1064 nm (PFCnDs-1064) and 760 nm (PFCnDs-760), enabling multiplex PA imaging and independent activation. Both species were synthesized with a lipid shell. (**B**) Schematic diagram of the experimental intracerebral delivery protocol. (B-1) Mice received a retro-orbital injection of PFCnDs-1064. (B-2) Mice received external laser stimulation at a wavelength of 1064 nm through the skull on the right side of the head. (B-3) Mice received an intra-jugular injection of PFCnDs-760. (**C**) Schematic diagram of the intracerebral delivery approach mechanism. (C-1) Intravenously injected PFCnDs-1064 flow into cerebral microvessels and approach the BBB. (C-2) Laser-stimulation induces PFCnDs-1064 cavitation and local BBB opening. (C-3) Intravenously injected PFCnDs enter brain tissue through the opened BBB

Building on the unique capabilities of PFCnDs to penetrate the blood-brain barrier and provide distinctive contrast mechanisms, we developed a strategy to facilitate intracerebral drug delivery tracking and transcranial US/PA imaging. Our approach utilizes PFCnDs to first induce localized BBB opening, allowing them to then extravasate through the BBB and subsequently generate contrast for sensitive detection and monitoring through the skull via US/PA imaging modes. Specifically, we engineered two distinct PFCnDs tailored for their specialized functions (Scheme 1A): For BBB opening, nanodroplets were constructed with perfluorohexane (PFH) (boiling temperature: 56 °C), which can recondense at normal body temperature (37 °C in humans), making perfluorohexane nanodroplets (PFHnDs) suitable for functions requiring oscillatory behavior [17, 18]. For CEUS imaging, nanodroplets were constructed with perfluoropentane (PFP) (boiling temperature: 28 °C). By having a boiling point lower than body temperature, perfluoropentane nanodroplets (PFPnDs) exhibit a low vaporization threshold and maintain a microbubble state after initial vaporization. Therefore, they provide persistent US contrast, which is ideal for CEUS imaging [11, 19, 24–26]. The two nanodroplet species were loaded with different dyes having distinct optical absorption spectra to enable independent laser activation and multiplex PA contrast. Specifically, PFHnDs and PFPnDs were loaded with dyes having peak absorbances of 1064 nm (PFCnDs-1064) and 760 nm (PFCnDs-760), respectively. For the shell of both nanodroplet species, a phospholipid coating was selected for its biocompatibility, ligand binding ability, and stability after vaporization [13]. By carefully modulating the PFCnD design in this manner, we aimed to create a versatile contrast agent system capable of BBB opening, extravasation into brain tissue, and providing both CEUS and PA contrast through the skull for mapping their distribution.

To demonstrate the utility of the constructed nanodroplets for intracerebral delivery and imaging, we employed the following experimental strategy in mice (Scheme 1-B, C): First, PFCnDs-1064 were injected retro-orbitally (Scheme 1B-1), allowing them to circulate and reach the cerebral microvasculature (Scheme 1C-1). Soon after, noninvasive laser stimulation was applied to the right brain hemisphere to induce recurrent vaporization of PFCnDs-1064 within the cerebral microvasculature (Scheme 1B-2). It is hypothesized that the cavitation of laser-activated PFCnDs disrupts the tight junctions between the endothelial cells of the BBB, thus inducing local BBB opening (Scheme 1C-2) [17, 27]. Subsequently, PFCnDs-760 were administered intravenously (Scheme 1B-3), enabling PFCnDs to penetrate the brain parenchyma through the disrupted BBB region (Scheme 1C-3). Four hours later, in vivo, transcranial US/PA imaging was performed to visualize the intracerebral nanodroplets. The in vivo imaging session used PA imaging wavelengths of 760 nm and 1064 nm. PFCnDs-760 were then detected and localized via PFCnD-mediated CEUS imaging. In addition, we demonstrated the ability of this system to characterize the three-dimensional distribution of nanodroplets in the brain through ex vivo, volumetric US/PA imaging. We also compared US/PA imaging data to immunohistochemical images of brain sections after PFCnD-induced BBB opening to validate the precision and accuracy of our approach.

## Materials and methods

### Synthesis of PFCnDs

Two PFCnD species were synthesized with a lipid shell composed of 1,2-distearoyl-sn-glycero-3-phosphoethanolamine-N-[amino(polyethylene glycol)-2000] (DSPE-PEG (2000), 25 mg/mL, Avanti Polar Lipids, Inc.) and 1,2-distearoyl-sn-glycero-3-phosphocholine (18:0 PC (DSPC), 25 mg/mL, Avanti Polar Lipids). PFCnDs-1064 were synthesized with PFH (FluoroMed, L.P.) and IR-1048 dye (Sigma‒Aldrich), which has a peak optical absorbance of 1064 nm. PFCnDs-760 were synthesized with PFP (FluoroMed, L.P.) and Epolight 9151 dye (Epolin, Inc.), which has a peak optical absorbance of 760 nm.

The following synthesis steps were performed twice, once for each PFCnD species. To create the lipid shell, 40 µL of DSPE-PEG (2000) and 8 µL of 18:0 PC (DSPC) were added to a 10 mL pear-shaped flask. An IR-1048 dye solution or Epolight 9151 dye solution, depending on the PFCnD species, was prepared in chloroform (1 mg/mL), and 200 µL was added to the flask. Another 1 mL of chloroform was added to the flask to create a smooth lipid cake. A rotary evaporator (Rotovapor, Büchi) was used at reduced pressure to remove the chloroform from the flask, leaving a dye-coated lipid cake. The solution was placed in a 7 mL scintillation vial, and 150 µL of PFH or PFP, depending on the PFCnD species, was added. The vial was vortexed for 10 s (Vortex Mixer, Fisher Scientific) and sonicated for five minutes in an ice-cold water bath (VWR, 180 W). The PFCnD solution was transferred to a 2 mL centrifuge tube and centrifuged for 60 s in a mini-centrifuge (Mini-Spin, Eppendorf) to remove excess PFC and dye. The supernatant was transferred to a new 2 mL centrifuge tube, and the pellet was discarded. Prior to in vivo experiments, the PFCnD solutions were left under UV light for 30 min for sterilization.

### Characterization of PFCnDs

Nanodroplet size and zeta potential were measured in phosphate-buffered saline (pH 7.4) using a dynamic light scattering instrument (DLS, Zetasizer Nano ZS, Malvern Instruments Ltd.). Nanodroplets were measured at room temperature (25^o^C). To understand the behavior of the nanodroplets after laser stimulation, the nanodroplet’s size were measured after in vitro laser stimulation with 200 and 1,200 pulses (Supplementary Figure S1 A-B). To understand the effect of physiological temperature on the nanodroplet’s size, DLS measurements were performed after heating the nanodroplets solutions to 37^o^C (Supplementary Figure S1 C-D). A spectrophotometer (Evolution 220, Thermo Scientific) was used to measure the optical absorption spectra of PFCnDs-760 and PFCnDs-1064. To remove the spectrophotometer measurement bias associated with the size of the nanodroplets, blank versions of the PFHnDs and PFPnDs were synthesized as described earlier but without adding dye and then measured by a spectrophotometer. The absorption spectra of the blank PFHnDs and PFPnDs were subtracted from the spectra of PFCnDs-1064 and PFCnDs-760, respectively.

### PFCnD-induced BBB opening and nanodroplet delivery

All animal studies were conducted under the protocol approved by the Institutional Animal Care and Use Committee at the Georgia Institute of Technology. Sustained released buprenorphine (IP, 0.8 mg/kg) was administered to each animal (BALB/c mouse, Jax) prior to anesthesia. Mice were anesthetized using a combination of isoflurane (2%, Henry Schein) and medical air (0.6 L/min, Airgas). Mice were stereotaxed in the prone position on a heating pad (Stoelting Co.). Hair from the scalp was removed through shaving and depilatory cream. Proparacaine (0.5%, Henry Schein) was applied to the eyes.

Next, the mice received a retro-orbital injection of 70 µL of PFCnDs-1064 (~ 10^8^ droplets) with a co-injection of 50 µL of 3% w/v sterile filtered Evans Blue (EB) (Sigma-Aldrich). To apply laser stimulation to the right side of the brain (when viewed rostral to caudal), animals were positioned underneath an unfocused 1.5 mm core diameter optical fiber (0.39 NA, Thorlabs, Inc.). Laser pulses were generated by an Nd: YAG laser (10 Hz pulse repetition rate, 5–7 ns pulse length, Vibrant, OPOTEK Inc.) at a wavelength of 1064 nm. Animals (*n* = 5) received laser stimulation at a laser fluence of 38 mJ/cm² for 600 pulses (t = 60 s) to prevent laser damage [18]. The ANSI standard for 1064 nm laser irradiation of the skin is 100 mJ/cm^2^ [28].

Following the PFCnD-induced BBB opening procedure, mice, still anesthetized, were removed from the stereotax and placed in the supine position. A jugular injection of 75 µL of PFCnDs-760 was administered, and the mice were allowed to recover while under monitoring for gross behavioral damage.

### In vivo, transcranial US/PA imaging

In vivo US/PA imaging was performed four hours after PFCnD-induced BBB opening to maximize the amount of nanodroplets that entered the brain, as transient BBB opening likely ends within four hours [29, 30]. The animals were anesthetized in the same way as before. Mice were imaged with a US/PA imaging system (Vevo LAZR, FUJIFILM VisualSonics, Inc.) using a 21 MHz ultrasound and photoacoustic imaging probe (LZ-250, FUJIFILM VisualSonics, Inc.) with a custom-built fiber jacket used to increase the US/PA system’s laser focus depth from 9 to 11 mm to 11–13 mm. Noninvasive imaging was performed through the skull; the skull and the scalp were left intact. The system’s Nd: YAG laser (20 Hz, 5–7 ns pulse length) was operated at two wavelengths. The animals were first imaged at 760 nm with a laser fluence of 22 mJ/cm^2,^ followed by imaging at 1064 nm and a laser fluence of 24 mJ/cm^2^. The ANSI standard for 760 nm laser irradiation of the skin is 26.4 mJ/cm^2^ [28]. The images were collected in a coronal orientation. A time-series of ultrasound and photoacoustic images were acquired in an alternating sequence at 5 frames/second.

### Ex vivo, volumetric US/PA imaging

To characterize the three-dimensional distribution of PFCnDs in the brain, volumetric US/PA imaging was conducted on a brain excised from a mouse treated with PFCnD-induced BBB opening.

After in vivo imaging, the animals were euthanized via an IP Euthatal injection (150 mg/kg) followed by perfusion with 1X PBS (pH 6.8) and 4% paraformaldehyde (PFA). Next, the animal’s heads were extracted and postfixed overnight in 4% PFA solution. After 24 h, the brains were excised and placed on top of an 8% gelatin base in a container filled with degassed water. Samples were imaged using a US/PA imaging system (Vevo LAZR, FUJIFILM VisualSonics, Inc.) with a 40 MHz ultrasound and photoacoustic imaging probe (LZ-550, FUJIFILM VisualSonics, Inc.). The tunable Nd: YAG laser (20 Hz, 5–7 ns pulse length) used in the US/PA imaging system was operated at two wavelengths. The brains were imaged at 760 nm with a laser fluence of 22 mJ/cm^2^ and at 1064 nm with a laser fluence of 24 mJ/cm^2^. Coronal 3D US/PA images at each wavelength were acquired with a distance step size of 0.102 mm for a total of approximately 14 mm.

### US/PA image processing

In vivo and ex vivo US/PA images were processed in MATLAB (MathWorks, Inc.). For PA signals, thresholding was applied to remove signals from endogenous chromophores. After thresholding, the PA signals were normalized such that each image had a dynamic range of 0 to 255. The ex vivo US/PA images were also processed and reconstructed with AMIRA (Thermo Scientific) to generate volumetric whole-brain images. Volumetric US/PA images were captured as a transverse view and corner cut view of the treated side of the brain.

### PFCnDs detection and localization

The in vivo US/PA imaging data was further processed to detect and localize PFCnDs-760 that have been delivered the brain. The repeated laser pulses for PA imaging at 760 nm also served to induce vaporization of PFCnDs-760. The time-series of US images captured the permanent transition of the PFCnDs-760 population into microbubbles. The time-series US imaging data was processed using a previously developed PFPnD detection algorithm that leverages the nanodroplet’s Boolean contrast abilities [23, 24]. In summary, we performed pixel-wise linear regressions on the US signals over time. The PFCnDs-760 signal was isolated from the background signal by evaluating each pixel’s linear slope. Pixels with US signal increases above a certain threshold were classified as containing PFCnDs-760. The PFCnDs-760-detected image is displayed over a coregistered ultrasound image, thus highlighting the location of PFCnDs-760 within the image. All pixels outside of the brain were omitted.

### Histology and immunohistochemistry

To confirm the efficacy of laser-activated PFCnDs for localized BBB opening and the accuracy of nanodroplet tracking, immunohistochemical analysis was performed. After ex vivo, volumetric US/PA imaging, the brains were transferred to a solution of 30% sucrose and stored at 4 °C for five days. The brains were snap frozen and mounted to a cryostat (Leica CM 1860, Leica Biosystems). To locate the area of BBB opening within the brain, Evans Blue was co-injected with PFCnDs-760. Coarse brain sections were cut in the rostral to caudal direction until Evans Blue was visually detected. From this point, 20 μm coronal sections were cut, collected, and analyzed, spanning a total region of 0–0.70 mm within the brain. Immunohistochemistry (IHC) was performed using goat anti-mouse IgG G (H + L) tagged with Alexa Fluor 488 (Invitrogen), and 4’,6-diamidino-2-phenylindole (DAPI, Invitrogen). IgG is a large, endogenous protein that cannot enter the brain unless BBB opening had occurred, making it an indicator of the spatial distribution of BBB opening [9, 31]. Pictomicrographs were captured with a ZEISS Laser Scanning Confocal Microscope 700 (ZEISS Group).

## Results

### Characterization of PFCnDs

**Fig. 1:**
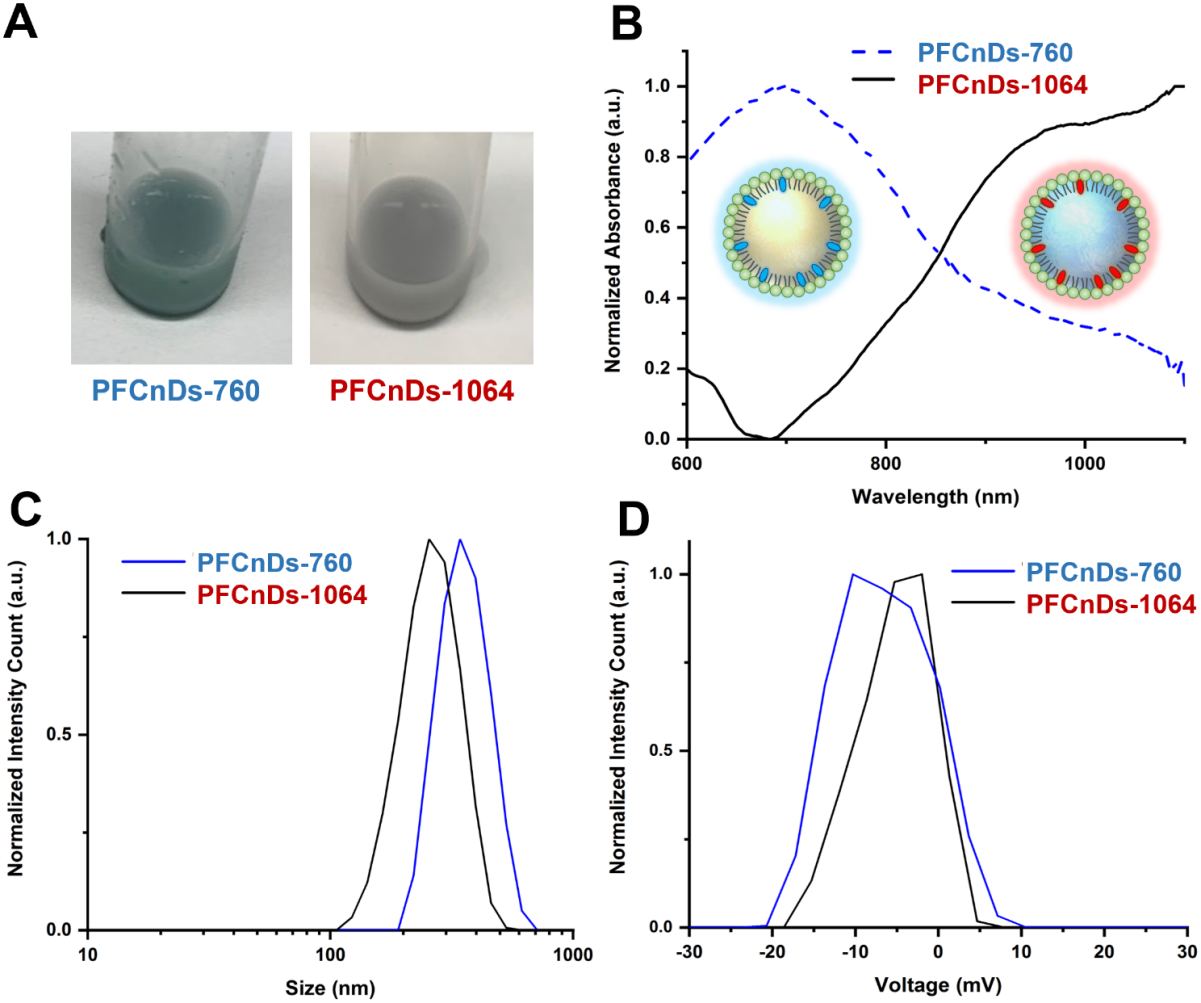
Characterization of two PFCnDs species for BBB opening and contrast-enhanced imaging. (**A**) Photographs showing the visual differences in solutions of PFCnDs-760 and PFCnDs-1064. (**B**) The UV‒VIS-NIR absorption spectra of PFCnDs-760 and PFCnDs-1064, measured using a spectrophotometer. The (**C**) size distributions and (**D**) Zeta potentials of PFCnDs-760 and PFCnDs-1064, measured by a Zetasizer Nano ZS

Two PFCnDs species were synthesized and loaded with dyes having a peak optical absorbance of 760 nm (PFCnDs-760) or 1064 nm (PFCnDS-1064) (Fig. 1A). Analysis of the optical absorption spectra indicated that each nanodroplet species had distinct spectra and unique peak absorbances, thus enabling independent activation and multiplex PA imaging of the two nanodroplet species (Fig. 1B). The absorption spectra of the nanodroplets corresponded to the absorption spectra of the dyes used for PFCnD production, Epolight 9151 (Epolin, New Jersey, USA) for PFCnDs-760 and IR-1048 (Sigma‒Aldrich, Missouri, USA) for PFCnDs-1064.

Size analysis revealed that the peak diameters of PFCnDs-760 and PFCnDs-1064 were approximately 342 nm and 255 nm, respectively (Fig. 1C). A submicron size is crucial for penetration past the opened BBB. The peak zeta potentials for PFCnDs-760 and PFCnDs-1064 were of the same sign, with measured peaks of -10.3 mV and − 1.98 mV, respectively (Fig. 1D).

### In vivo, contrast-enhanced US/PA imaging of the brain

**Fig. 2:**
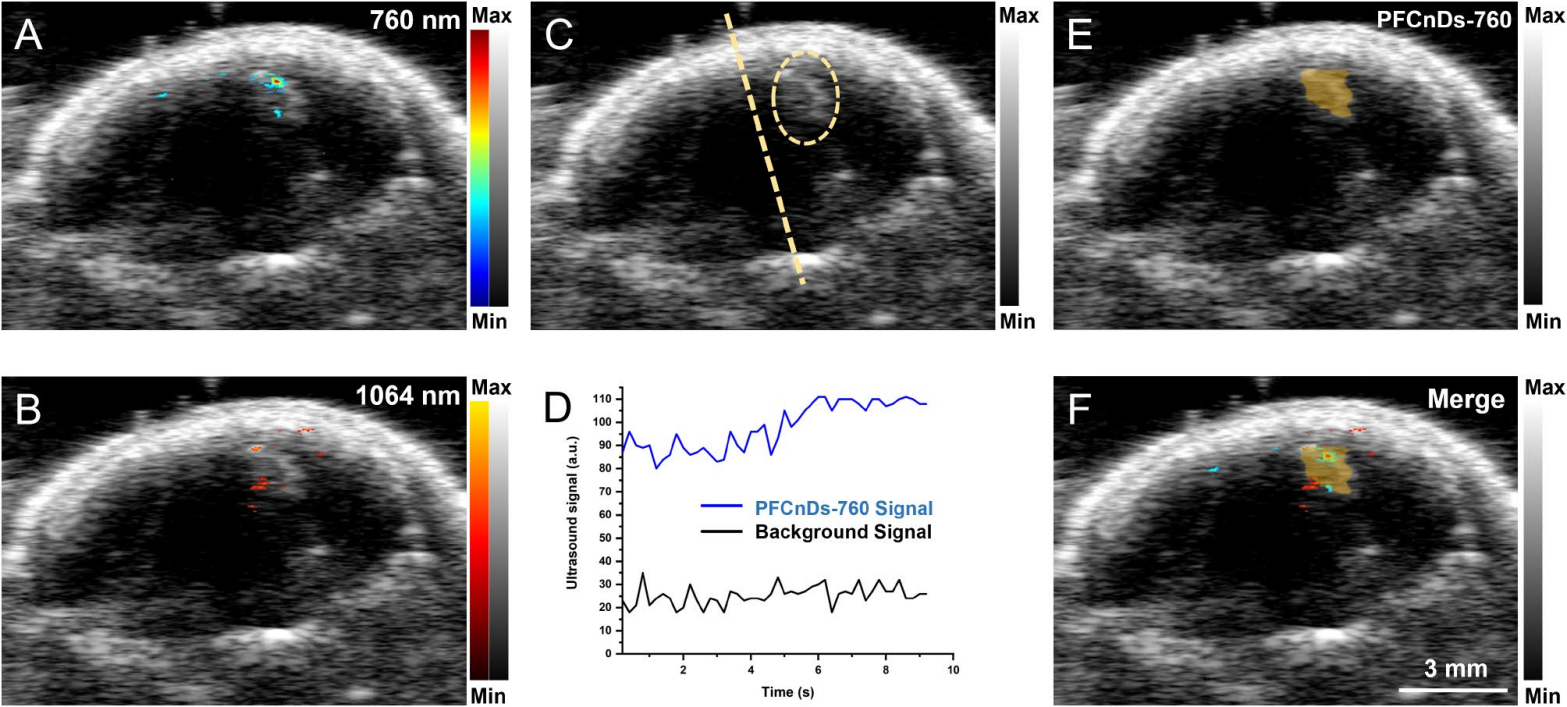
US/PA imaging data of a mouse brain treated with the PFCnDs-based intracerebral delivery approach. All images have a coronal orientation and are viewed in the caudal direction. In vivo, transcranial US/PA imaging was performed four hours after nanodroplet delivery without removing the skull or scalp. (**A**, **B**) US/PA images, acquired at PA imaging wavelengths of 760 nm and 1064 nm (color bars). Thresholding was applied to remove signals from endogenous chromophores. US imaging (grayscale) generated an anatomical map of the head, skull, and brain. US/PA images of the head show 760 nm and 1064 nm signals localized toward the treated (right) side of the head. (**C**) US B-mode image, acquired after laser-activation of PFCnDs-760. The image shows a bright, echogenic region on the treated side of the head (circled). (**D**) Time-series US intensity signals, during the application of 760 nm wavelength laser stimulation. The PFCnDs-760 and background signals were acquired as a mean of the pixels of regions on the treated (right) and untreated (left) sides of the brain, respectively. The PFCnDs-760 signal increased in response to laser stimulation, while the background signal remained constant. (**E**) PFCnDs-760-detected image acquired using PFCnD-mediated CEUS imaging and a PFPnD detection algorithm. The algorithm highlighted US B-mode image pixels (yellow) based on their response to laser stimulation. The PFCnDs-760-detected is overlaid on the US B-mode image. The PFCnDs-760-detected signal was localized to the treated side of the brain. (**F**) A merged view image of all measured signals. The image shows an overlapping distribution of PA, US, and PFCnDs-760-detected signals

US B-mode imaging generated an anatomical, coronal view of the brain, depicted as a dark region surrounded by a bright, round border corresponding to the skull as viewed (Fig. 2A, B, C, E, F). PA imaging at laser wavelengths of 760 nm and 1064 nm (Fig. 2A, B) revealed high PA contrast, produced by PFCnDs-760 (Fig. 2A) and PFCnDs-1064, demonstrating that the nanodroplets provided multiplex PA signals (Fig. 2B). For PFCnD-induced BBB opening, external 1064 nm laser stimulation was applied only to the right side of the mouse’s head; therefore, the left side of the brain was used as a control. The overlapping spatial distribution of the 760 nm and 1064 nm PA signals in a localized region on the treated (right) side of the brain, with little signal on the control side, indicated precise delivery of the nanodroplets.

After 760 nm wavelength laser stimulation, B-mode images revealed an echogenic region, hypothesized to contain PFCnDs-760, on the treated (right) side of the brain (Fig. 2C). In contrast, the untreated (left) side of the brain showed hypoechoic or background signal. When comparing the results across the mouse cohort, we observed similar isolated echogenic regions on the treated side of the brain (Supplemental Figure S2, A-C). A Welch Two Sample T-test revealed a significant difference (p-value = 0.007346) between the mean US intensity on the treated against the untreated side of the brain for the experimental cohort, indicating the feasibility of reproducibility of the technique (Supplemental Figure S2, D).

The time-series US intensity signal for a region of interest within the echogenic region on the treated side of the brain showed an increase over time indicating a causal response of the PFCnDs-760 signal to 760 nm laser stimulation (Fig. 2D). The background, US intensity signal from a region of interest on the untreated side of the brain was constant, having no response to 760 nm laser stimulation. The PFPnD detection algorithm was applied to the B-mode images to generate a high-confidence prediction of nanodroplet presence within each image pixel; the PFCnDs-760-detected pixels are highlighted in yellow (Fig. 2E). The PFCnDs-760-detected image showed a localized signal within the treated side of the brain, and a merged view of all the measured and computed signals showed a spatial overlap between all the signals (Fig. 2F). The area detected by PFCnDs-760 was wider than that indicated by the PA signals and aligned with the echogenic region observed in the unprocessed B-mode US image. This detection based on PFCnDs-760 signals offers a robust indication of nanodroplet location, derived from a causal analysis of the nanodroplet response to laser stimulation. Furthermore, the continuous and outlier-free nature of the PFCnDs-760-detected area illustrates a localized region of blood-brain barrier (BBB) opening with high confidence.

### Distribution of intracerebral PFCnDs via volumetric US/PA imaging

**Fig. 3:**
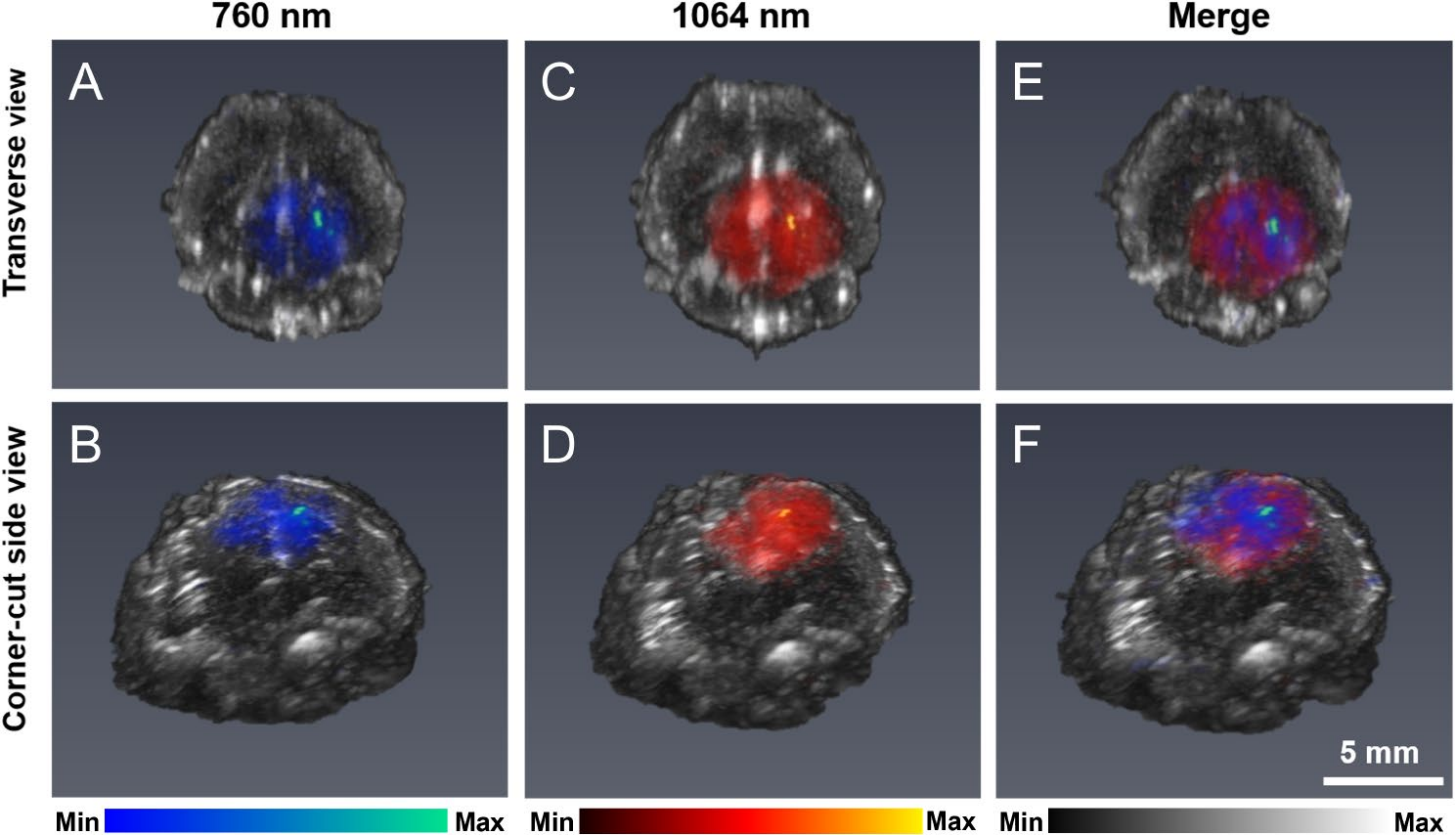
Volumetric US/PA images of an excised brain from a mouse treated with the intracerebral imaging and delivery approach. The mouse received intravenous injection of PFCnDs-760 and PFCnDs-1064 and was treated with PFCnD-induced BBB opening. The animal was euthanized four hours after nanodroplet injection. PA images used imaging wavelengths of (**A**, **B**) 760 nm and (**C**, **D**) 1064 nm. (**E**, **F**). A merged image of the signals acquired at both wavelengths is shown. Images show the localization of PFCnDs to the treated (right) side of the brain. The image perspectives include transverse (**A**, **C**, **E**) and corner-cut (**B**, **D**, **F**) views of the treated (right) side of the brain

Volumetric US imaging generated an anatomical map of the brain tissue as a three-dimensional image displayed in grayscale (Fig. 3). Volumetric PA imaging using 760 nm (blue) and 1064 nm (red) imaging wavelengths showed high-sensitivity contrast from 760 nm and 1064 nm absorbing photoabsorbers within the brain tissue (Fig. 3). The excised brains were fixed, and blood was flushed via transcardial perfusion, suggesting that strong PA signals originated from within the brain tissue rather than from the vasculature. These signals showed three-dimensional localization of the PA signal at both imaging wavelengths on the laser stimulation-treated (right) side of the brain, indicating the entry of PFCnDs into the brain tissue. In addition, the 1064 nm and 760 nm signals showed high spatial overlap, demonstrating that the PA signal sources were colocalized (Fig. 3E-F).

### Immunohistochemical validation of intracerebral delivery and imaging approach

**Fig. 4:**
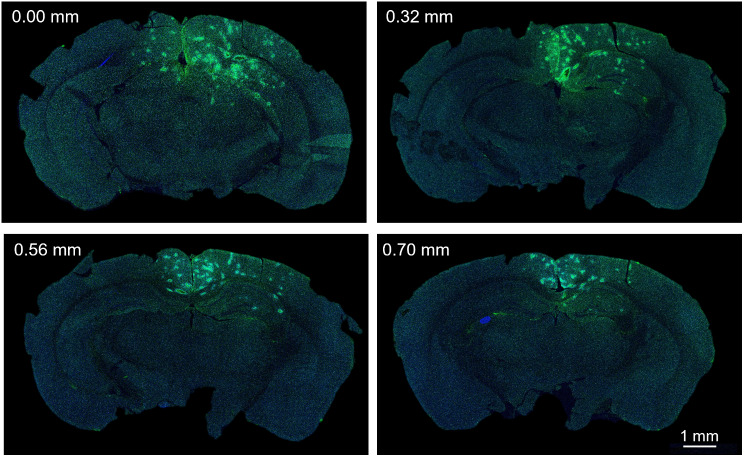
Immunohistological analysis of mouse brains treated with PFCnD-induced BBB opening. Images are in coronal orientation and labeled by sectioning distance. Sections were stained with anti-IgG (green) and DAPI (blue). Localized IgG fluorescence was present on the treated (right) side of the brain. The untreated (left) side of the brain shows little to no IgG fluorescence

Fluorescence microscopy revealed an IgG population (green) within the treated (right) side of the brain, verifying the success of PFCnD-induced BBB opening (Fig. 4). The intensity of IgG fluorescence is specifically high in the superior neocortex, extending deep into the hippocampus formation. In addition, there was little to no IgG fluorescence on the far-left side of the brain, indicating that BBB opening was localized. This fluorescence pattern was consistent across the various sectioning distances (Fig. 4A-E).

## Discussion

Imaging investigations demonstrated the successful deployment of PFCnDs for intracerebral delivery and imaging. First, the visualization of nanodroplets that penetrated brain tissue was achieved through noninvasive, in vivo, and transcranial US/PA imaging (Fig. 2). The nanodroplet species could be differentiated using multiplex US/PA imaging (Figs. 2A and B and 3A-F). The presence of nanodroplets was confirmed using their Boolean contrast abilities and US imaging coupled with laser stimulation (Fig. 2C-E). The stability of the PFCnDs allowed them to produce dynamic contrast and thus enable contrast-enhanced imaging at least four hours after injection. Next, volumetric US/PA imaging provided a three-dimensional characterization of the spatial distribution of nanoparticles delivered to the brain while still providing multiplexed information (Fig. 3). Future investigations could explore spectroscopic PA analysis and spectral unmixing to broaden the approach’s detection capabilities, enhance the sensitivity of nanodroplet tracking, and differentiate among more than two nanodroplet species [32]. In addition, contrast harmonic imaging could be employed to increase the sensitivity of PFPnD detection.

Histological studies demonstrated the effectiveness and precision of the intracerebral delivery system. Immunohistology employing anti-IgG demonstrated the entry of large molecules into the brain, thus confirming that BBB opening had been achieved (Fig. 4). The localization of IgG to the treated (right) side of the brain demonstrated that the platform can use directed laser light to perform targeted and localized intracerebral delivery. In addition, the spatial distribution of IgG matched the localization of the nanodroplets within the brain, as determined by US/PA imaging. Therefore, US/PA imaging can accurately track intracerebral nanodroplets and can report delivery accuracy and precision. A limitation of this study was the absence of histological data for PFCnDs, a gap that could be addressed in future studies by incorporating fluorescent labels for nanodroplets.

When comparing the PA imaging results between the in vivo and ex vivo experiments, the latter shows a larger region of high contrast PA signal on the treated side of the brain for both wavelengths. Ex vivo PA imaging was performed after removing the skull and skin, both of which are strong optical scatterers. Perfusion also flushed out most of the blood, a potent endogenous absorber that produces background PA signal at near-infrared wavelengths [33].

Laser-activated PFCnDs offer unique advantages because of their function as intracerebral contrast and therapeutic agents. They offer dual US/PA contrast, contrast-enhanced and multiplex imaging capabilities; are synthesized with biocompatible FDA-approved components (expediting translation to clinical applications); and are easily modified with targeting moieties for in vivo neuroimaging. Moreover, PFCnDs can serve as carriers for localized delivery to brain tissue, with confirmation of intracerebral delivery achieved via validated, transcranial US/PA imaging of therapeutic PFCnDs [34]. By leveraging their phase change mechanism and cavitation-like behavior, laser-activated PFCnDs can be used for tumor cell destruction or enhancing the uptake of viral vectors for gene transduction [34–36]. Future applications of PFCnDs could facilitate simultaneous BBB opening, drug delivery, treatment monitoring, and other cavitation-based therapeutic interventions. Also, by binding PFCnDs to targeting ligands, modified PFCnDs could provide US/PA contrast for specific brain tissues and brain tumors. For example, cyclic[RGDyK] peptides, having affinity for tumor vasculature and glioblastoma, have been validated for delivery of therapeutic photoacoustic nanoparticles to brain tumors [16]. Thus, PFCnDs hold promise in clinical applications not only as contrast agents for US/PA neuroimaging and delivery vehicles but also as versatile platforms for multifaceted therapeutic applications.

In addition, the intracerebral delivery and imaging system could also facilitate future investigations into fundamental principles in drug delivery, including the characteristics of BBB opening mediated by FUS-stimulated microbubbles, or laser-activated PFCnDs. While the current study is limited to in vivo imaging at a single time point, tracking the extravasation of molecules across multiple time points could yield crucial insights into the spatial and temporal dynamics of molecule transport into the brain post-BBB opening, thereby contributing to advancements in drug development.

## Conclusion

The intracerebral delivery and imaging strategy presented here enabled the delivery of PFCnDs to the brain for transcranial US/PA imaging. This integrated system addresses critical challenges in intracerebral drug delivery, including the need for intracerebral delivery tracking tools and the limitations of transcranial US/PA imaging. Through PFCnD-induced BBB opening and owing to their small size, nanodroplets could be delivered to targeted regions of the brain. Within brain tissue, PFCnDs provided US/PA contrast and enabled contrast-enhanced imaging through noninvasive US/PA imaging. In addition, by incorporating optical triggers with distinct absorption spectra, transcranial multiplex US/PA was able to distinguish signal between two nanodroplet subpopulations. Due to their stability, small size, and various functionalities, PFCnDs are promising for use in intracerebral drug delivery and tracking.

### Electronic supplementary material

Below is the link to the electronic supplementary material.

Supplementary Material 1

Supplementary Material 2

Supplementary Material 3

## Data Availability

The datasets used and/or analyzed during the current study are available from the corresponding author upon reasonable request.
