# Supplementary material for: Laser-activated perfluorocarbon nanodroplets for intracerebral delivery and imaging via blood–brain barrier opening and contrast-enhanced imaging
Source: J Nanobiotechnology. 2024 Jun 21;22:356. doi: 10.1186/s12951-024-02601-6 (PMC11191388; doi:10.1186/s12951-024-02601-6)
Supplement: Supplementary file 1 — Supplementary Material 1 [file 12951_2024_2601_MOESM1_ESM.docx]

**Supplementary Information**

Laser-Activated Perfluorocarbon Nanodroplets for Intracerebral Delivery and Imaging via Blood–Brain Barrier Opening and Contrast-Enhanced Imaging

*Kristina A. Hallam^1,2^, Robert J. Nikolai^1^, Anamik Jhunjhunwala^1^, Stanislav Y. Emelianov^1,2, *^*

Affiliations:

*1. The Wallace H. Coulter Department of Biomedical Engineering, Georgia Institute of Technology and Emory University School of Medicine, Atlanta, GA, USA*

*2. School of Electrical and Computer Engineering, Georgia Institute of Technology, Atlanta, GA, USA*

Keywords:

*Photoacoustic imaging, Ultrasound imaging, Perfluorocarbon nanodroplets, Drug delivery, Neuroimaging, Contrast-enhanced imaging, Multiplex imaging, Blood‒brain barrier opening.*

Corresponding author: Stanislav Y. Emelianov

E-mail: [stas@gatech.edu](mailto:stas@gatech.edu)


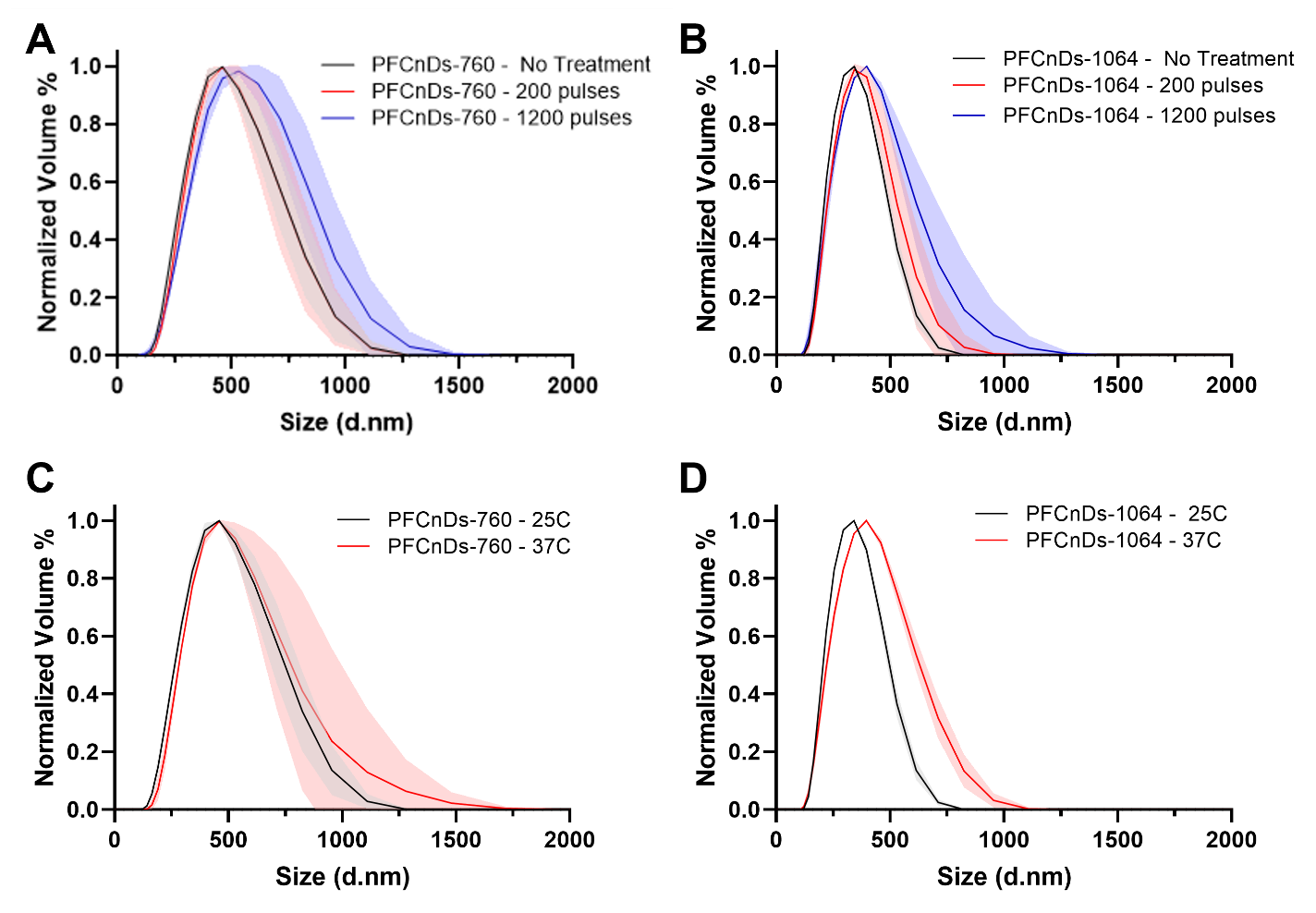


**Figure S1:** Volume-weighted hydrodynamic size distributions by volume measurements of nanodroplets. Plotted data are average of n=3 technical replicates. Solid lines indicate mean estimate and shaded bands show standard error. (A, B) PFCnDs-760 and PFCnDs-1064 were measured before and after laser stimulation treatment with 760 nm or 1064 nm laser light, respectively. Nanodroplets received either no treatment (black), 200 pulses of laser stimulation (red), or 1200 pulses of laser stimulation (blue). (C, D) PFCnDs-760 and PFCnDs-1064 were measured at 25^o^C (black) and 37^o^C (red).


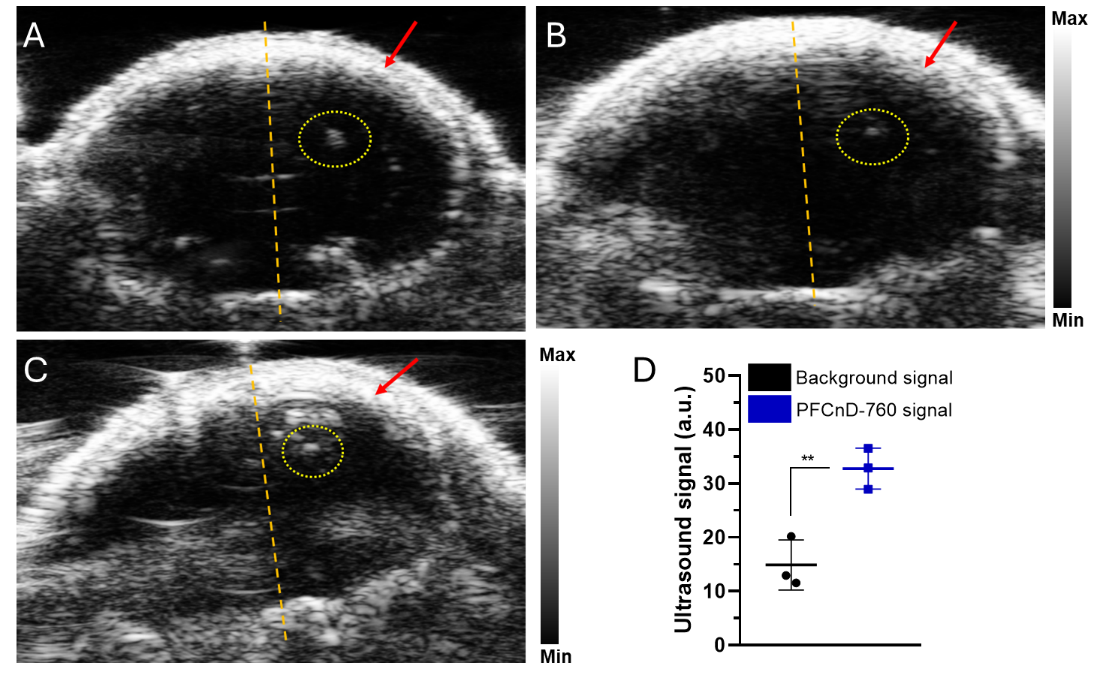


**Figure S2**: (A-C) US B-mode images of three biological replicates, acquired 4-hours after PFCnD-induced BBB opening and 760 nm laser-stimulation was applied to vaporize PFCnDs-760. The image shows a bright, echogenic region on the treated side of the head. To identify PFCnDs-760, the US signals from the echogenic region (circled in yellow dotted line) was compared to a region in the untreated side of the brain (region of same size taken from same depth but across the orange dashed line). (D) US signal intensity for the PFCnDs-760 (treated side) and the background (untreated side) of the brain (n=3). The statistical analysis revealed a significant difference in the ultrasound (US) signals between the laser-stimulated and non-stimulated sides of the brain (Welch Two Sample T-test, p = 0.007346, t = -5.1768, df = 3.8493).
