# Supplementary figures and images for: Laser-activated perfluorocarbon nanodroplets for intracerebral delivery and imaging via blood–brain barrier opening and contrast-enhanced imaging
Source: J Nanobiotechnology. 2024 Jun 21;22:356. doi: 10.1186/s12951-024-02601-6 (PMC11191388; doi:10.1186/s12951-024-02601-6)

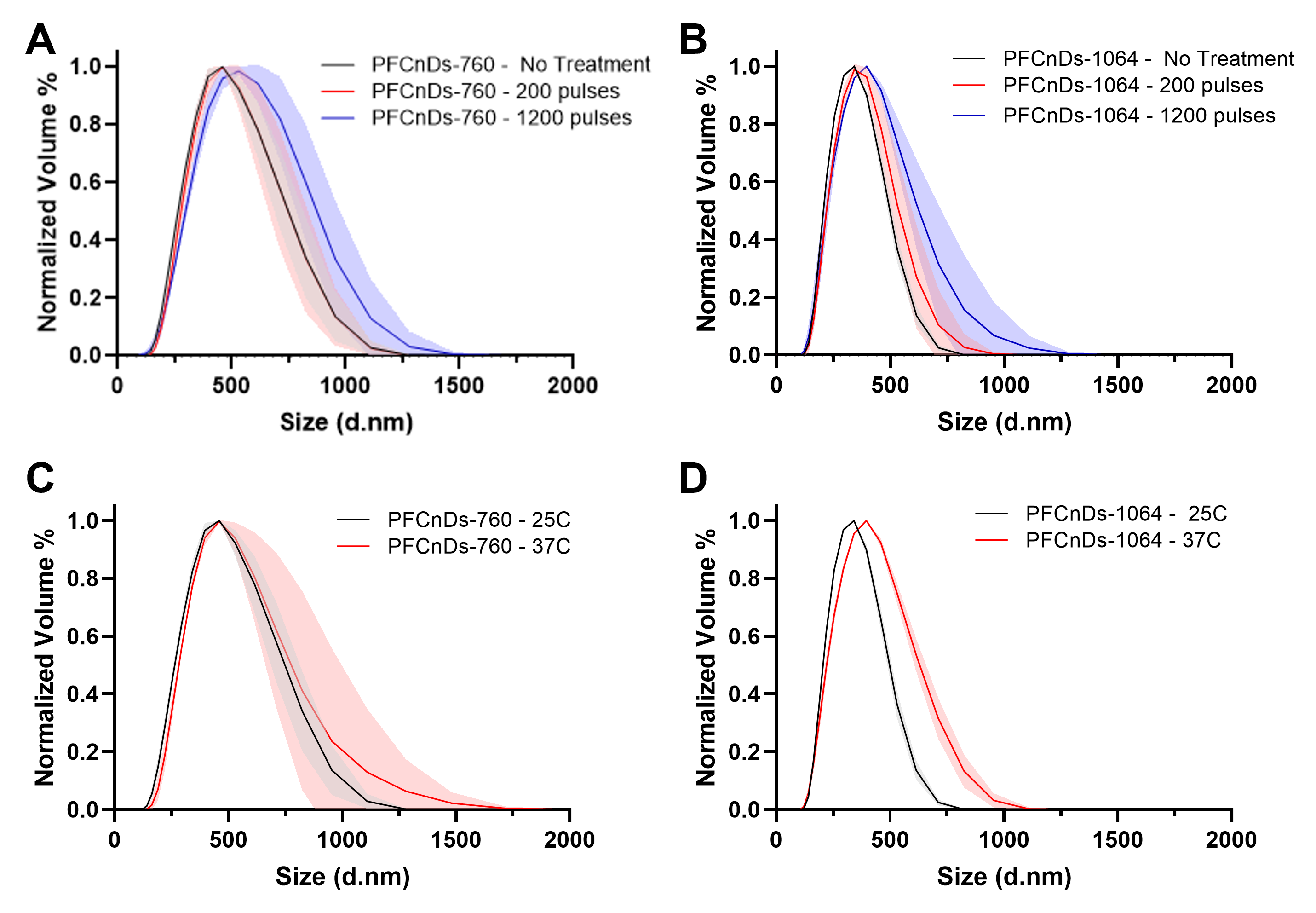

Supplement: Supplementary file 2 — Supplementary Material 2 [file 12951_2024_2601_MOESM2_ESM.tif]

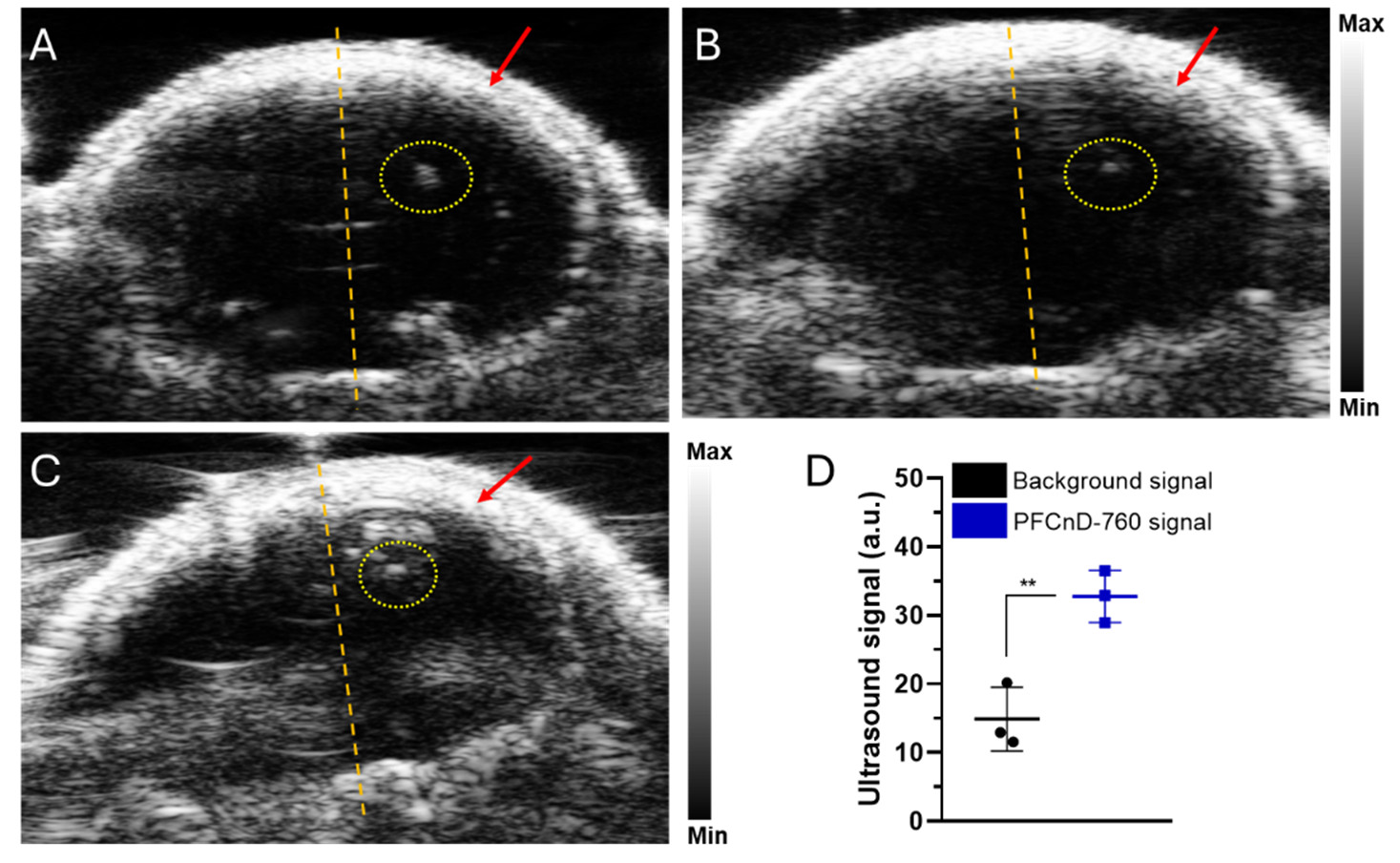

Supplement: Supplementary file 3 — Supplementary Material 3 [file 12951_2024_2601_MOESM3_ESM.tif]
